# Posttranslational Modifications of Smurfs: Emerging Regulation in Cancer

**DOI:** 10.3389/fonc.2020.610663

**Published:** 2021-02-22

**Authors:** Longtao Yang, Wenwen Zhou, Hui Lin

**Affiliations:** ^1^ Second Clinical Medical School, Nanchang University, Nanchang, China; ^2^ Department of Pathophysiology, School of Basic Medical Sciences, Nanchang University, Nanchang, China

**Keywords:** Smurfs, posttranslational modification (PTM), cancer, phosphorylation, ubiquitination

## Abstract

Smad ubiquitination regulatory factors (Smurfs) belong to the Nedd4 subfamily of HECT-type E3 ubiquitin ligases. Under normal situations, Smurfs are exactly managed by upstream regulators, and thereby strictly control tumor biological processes, including cell growth, differentiation, apoptosis, polarization, epithelial mesenchymal transition (EMT), and invasion. Disruption of Smurf activity has been implicated in cancer progression, and Smurf activity is controlled by a series of posttranslational modifications (PTMs), including phosphorylation, ubiquitination, neddylation, sumoylation, and methylation. The effect and function of Smurfs depend on PTMs and regulate biological processes. Specifically, these modifications regulate the functional expression of Smurfs by affecting protein degradation and protein interactions. In this review, we summarize the complexity and diversity of Smurf PTMs from biochemical and biological perspectives and highlight the understanding of their roles in cancer.

## Introduction

**Figure 1:**
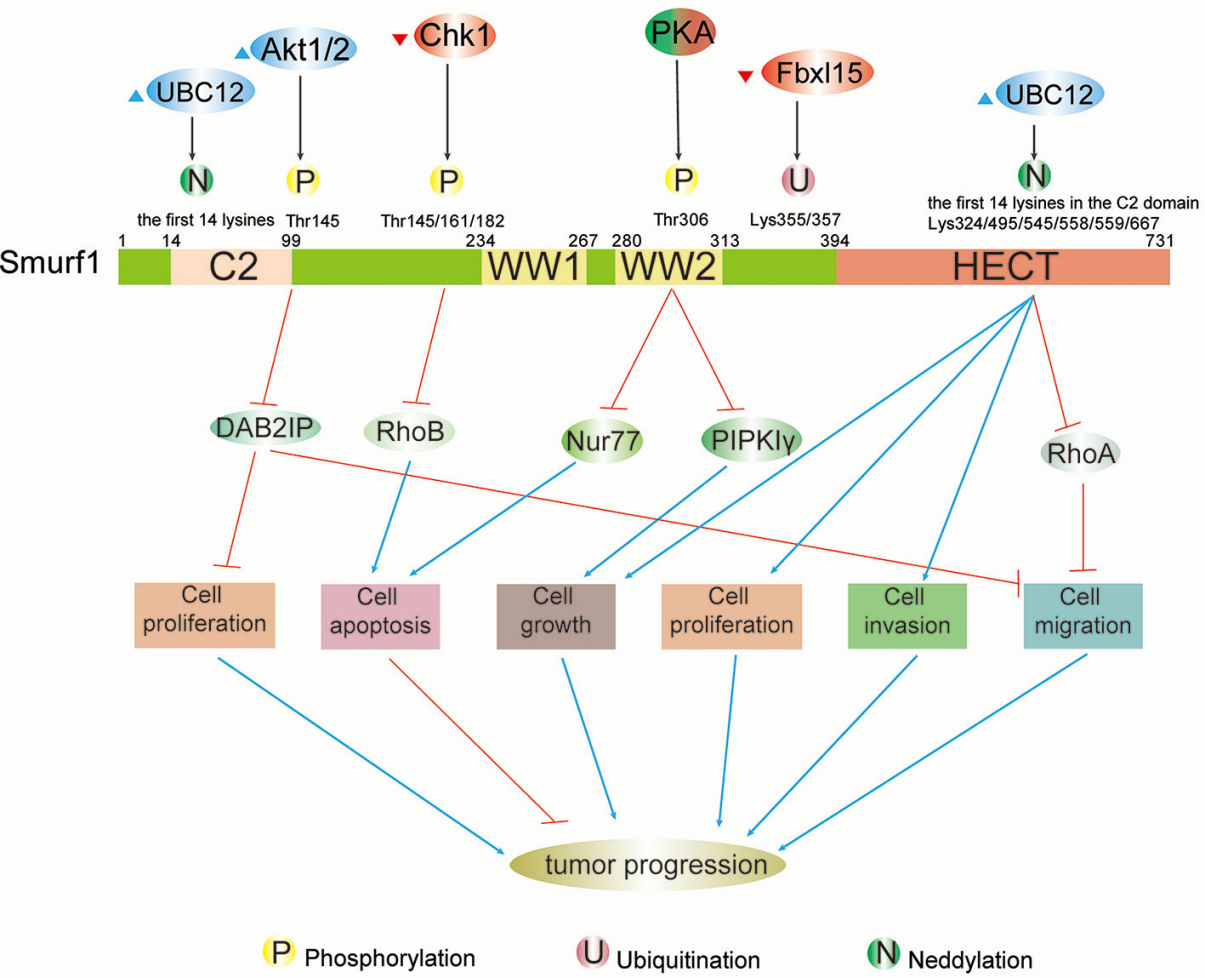
Schematic summary of modifications and functions of Smurf1 in cancers. Full-length Smurf1 is schematically depicted and includes an N-terminal C2 domain, two WW domains, and one C-terminal HECT domain. Posttranslational modifications occurring at specific locations on Smurf1 are indicated, including phosphorylation, ubiquitination, and neddylation. Phosphorylation: phosphorylation of Smurf1 by Akt1/2 increases degradation of DAB2IP, which promotes cell proliferation and migration. Phosphorylation by Chk1 downregulates Smurf1 and leads to the accumulation of RhoB, which facilitates apoptosis. Under different circumstances, PKA has a dual role in tumor progression that results in high protein levels of Nur77 and PIPKIγ that promote apoptosis and cell growth, respectively. Neddylation: neddylation of Smurf1 by UBC12 leads to cell migration by enhancing downregulation of RhoA, as well as cell growth, proliferation, and invasion. However, the detailed mechanisms of Smurf1 neddylation by UBC12 need to be further researched.

**Figure 2:**
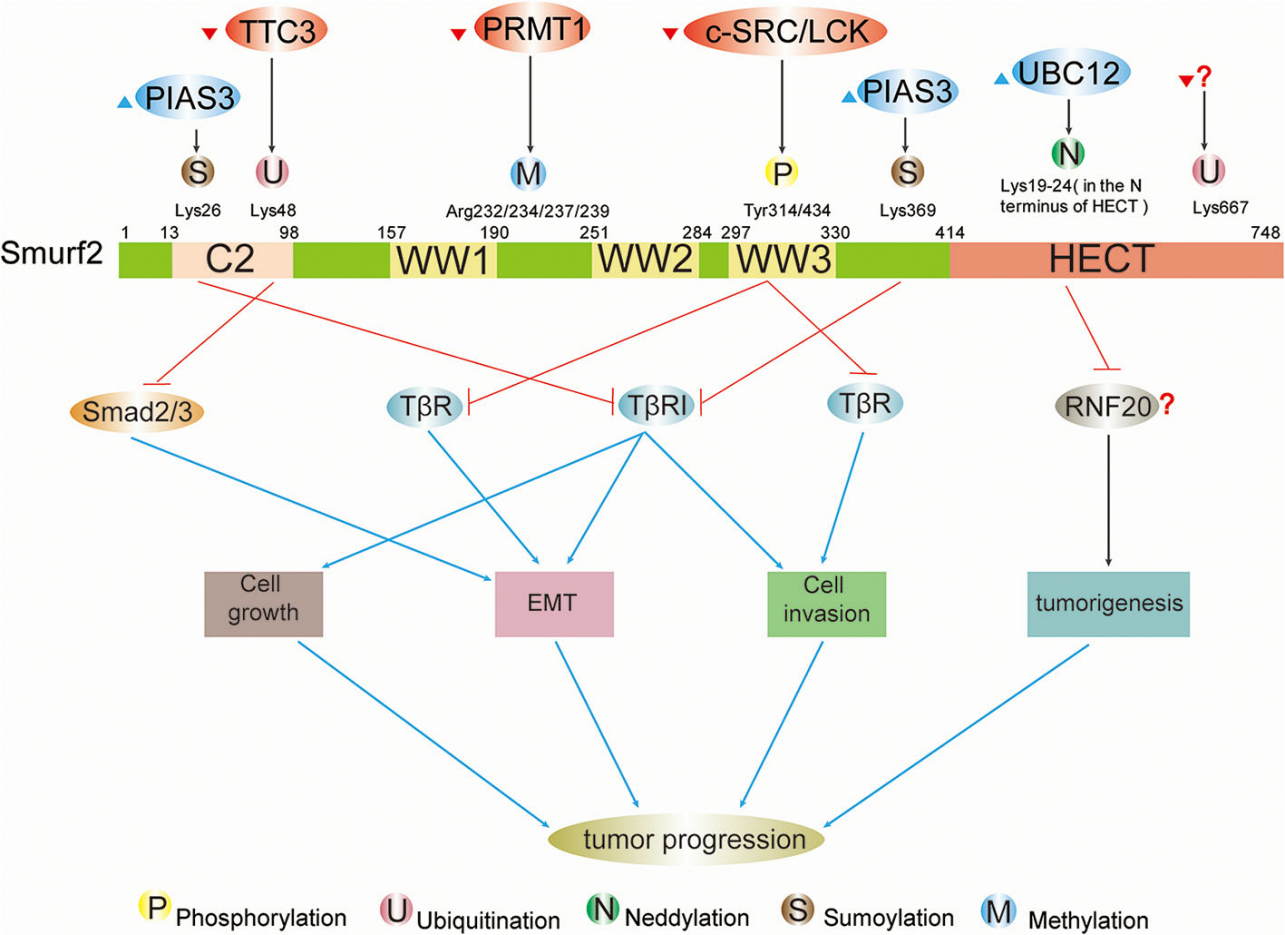
Schematic summary of upstream regulatory factors and modification sites and functions of Smurf2 in cancers. Full-length Smurf2 is schematically depicted and includes an N-terminal C2 domain, three WW domains, and one C-terminal HECT domain. Posttranslational modifications occurring at specific locations on Smurf2 are indicated, including phosphorylation, ubiquitination, neddylation, sumoylation, and methylation. Phosphorylation: phosphorylation by c-SRC/LCK downregulates Smurf2, resulting in stabilization of TβR and leading to EMT and cell invasion. Ubiquitination: ubiquitination event by TTC3 stabilizes Smad2/3 and facilitates EMT by degrading Smurf2. The protein that ubiquitinates Smurf2 at Lys667 is unknown. Neddylation: further study is needed to determine whether neddylated Smurf2 targets RNF20 for degradation and thus modulates tumorigenesis. Sumoylation: sumoylation of Smurf2 by PIAS3 facilitates Smurf2-mediated degradation of TβRI and suppresses tumor cell EMT, growth and invasion.

Smurfs regulate effectors in a host of signaling cascades (e.g., TGF-β, BMP, RAS, Wnt) (1). The function of Smurfs is to mediate mono- or poly-ubiquitination of substrates, which modulates the stability, abundance, and positioning of the protein (1). In fact, protein ubiquitination is a dynamic and multifaceted posttranslational modification that involves the control of nearly all physiological activities in eukaryotic cells (2). The diversity and complexity of regulatory mechanisms also require that Smurfs’ recognition and ubiquitination of substrates must be a highly specific and adjustable process. As is known to all, the specific amino acid sequence and spatial structure in biological element determine the interaction between proteins, which is closely related to their functions (3). Smurfs are no exception, containing multiple domains that match their functions. The molecular weight of Smurfs is measured by kilodalton (kD), which is a unit used for counting the algebraic sum of the atomic weights of all atoms in the molecule (4). Human Smurf1 contains an N-terminal C2 domain (14–99 kD) for phospholipids and Ca^2+^ binding (5), two WW domains (234–267 kD; 280–313 kD) primarily for interaction with PPXY or LPXY motifs and phospho-serine/threonine residues of substrates (6), as well as a C-terminal HECT domain (394–731 kD) for transferring ubiquitin from the catalytically active site Cys699 to substrates (7) (**Figure 1**). Human Smurf2 has one additional WW domain (297–330 kD) based on Smurf1, and its catalytically active site is Cys716 (8) (**Figure 2**).

Early on, it was found that Smurfs could guide the direction of tumor biological processes through TGF-β signaling. As E3 ligases, Smurfs are involved in the ubiquitination and degradation of TGF-β receptors, and the ubiquitination process involves crosstalk between Smurfs and Smad proteins (9). The Smurf1/2-Smad7 complex not only triggers proteasomal degradation of TβRI *via* ubiquitination but also blocks Smad3 complex formation by mono-ubiquitinating Smad3, both of which result in downregulation of the TGF-β signaling pathway (10, 11). TGF-β signaling is intimately correlated with cancer. However, its effects on cancers are not homogeneous; instead, it drives tumors toward two completely opposite outcomes at different stages. During the early stage of cancer, the TGF-β cascade inhibits cell proliferation and induces apoptosis (12); however, in advanced cancer, disordered TGF-β pathway signaling reverses that inhibition (13).

Numerous studies have found that in addition to relying on the TGF-β pathway, Smurfs also modulate tumor cell activities in a TGF-β cascade-independent manner, which broadens the diversity of Smurfs’ substrates. Due to their substrate diversity and specificity, Smurfs play a negative or positive role in tumor development. Specifically, as a tumor promoter, Smurf1/2, which is remarkably highly expressed in tumor cells, leads to poor prognosis. For example, Smurf1 boosts cell invasion by degrading tumor suppressors [e.g., Ras homolog gene family member A (RhoA), hPEM-2, DAB2 interacting protein (DAB2IP), Kindlin-2] and altering cellular localization of substrates [e.g., tumor necrosis factor receptor-associated factor 4 (TRAF4)] (14–18), and it inhibits p53/DNA damage-mediated apoptosis by degrading corresponding factors (e.g., ING2, RhoB, KLF2) and stabilizing MDM2 (19–22). Stabilization of Mad2 and Nedd9, mediated by Smurf2, facilitates the cell cycle (23, 24). However, Smurfs, when acting as tumor suppressors, inhibit tumor development. At the protein level, Smurf1 suppresses tumor progression *via* inhibition of cell proliferation, survival, and metastasis by degrading TRIB2, MCAM, and SRSF5, as well as by nonproteolytically regulating AXIN1 (25–28). Tumorigenesis is repressed by Smurf2 through its degradation of Id1 *via* ubiquitination and its facilitation of autophagosome-mediated lysosomal turnover of LaminA/progerin (29, 30). At the transcriptional levels, Smurf2 mediates degradation of transcriptional factors (e.g., KLF5, YY1), which blocks cell proliferation (31, 32), and decreases susceptibility to various cancers by maintaining genomic stability (33, 34). Furthermore, some of Smurfs’ special substrates [e.g., Skp1-cullin-1-F-box protein (SCF) ubiquitin ligase complex, ring finger protein 20 (RNF20)] have a wholly opposite role in tumor progression in different cell types and cellular contexts, which further broadens the complexity of Smurfs’ regulation of biological functions (33, 35–37).

In short, due to the dual role of Smurfs in tumors, along with the diversity and complexity of their downstream regulatory mechanisms, Smurfs seem to act as decisive regulators among signaling pathways, but they do not act alone in cancer cells. To a certain extent, Smurfs themselves are largely affected by numerous upstream factors. With respect to protein levels, some adaptors (e.g., Smad6/7) interact with Smurf1/2, enhancing its ubiquitin ligase activity toward proteins, while several regulatory elements (e.g., PTPN3) block the binding of Smurfs to their substrates, hindering substrate degradation (1). In addition to the effects of noncovalent binding, in recent years a host of studies have investigated important posttranslational modifications of Smurfs, including phosphorylation, ubiquitination, neddylation, sumoylation, and methylation, and the significance of PTMs has become increasingly prominent in cancers (**Table 1**). PTMs regulate Smurfs in a highly specific manner, providing unique potential targets with high specificity and accuracy. In this context, summarizing upstream regulatory factors of Smurfs and the effects of Smurf modifications on their substrates is significant for an in-depth analysis of Smurfs’ biological functions and tumor-targeted therapies.

**Table 1:** Published posttranslational modifications and thus functions of Smurfs.

PTM	Residue	Enzyme	Function	Reference
Phosphorylation	Smurf1 Thr306	PKA	Inhibit Smurf1 ubiquitin ligase activity: reduce degradation of PIPKIγ	(38)
	Smurf1 Thr306	PKA	Promote Smurf1-mediated unconventional ubiquitination of substrate: inhibit degradation of Nur77	(39)
	Smurf1 Thr306	PKA	Alter Smurf1 affinity for substrates: inhibit degradation of Par6; increase degradation of RhoA	(40)
	Smurf1 Thr145	Akt1/2	Increase protein level of Smurf1: degrade DAB2IP *via* ubiquitination	(16)
	Smurf1 Thr145/161/182	Chk1	Promote ubiquitination and degradation of Smurf1: inhibit degradation of RhoB	(20)
	Smurf1 Thr145/161/182	IRAK2	Promote ubiquitination and degradation of Smurf1: result in altered cascade of ER effectors	(41)
	Smurf2 Tyr314/434	c-SRC、LCK	Inhibit Smurf2 ubiquitin ligase activity: promote TGF-β signaling by suppressing degradation of TβR	(42)
Ubiquitination	unknown	Smurf2	Promote ubiquitination and degradation of Smurf1: affect mechanism independent on TGF-β signaling	(43)
	unknown	CKIP-1	Promote ubiquitination and degradation of Smurf1: inhibit degradation of p53	(44)
	Smurf1 K355/357	Fbxl15	Promote ubiquitination and degradation of Smurf1: promote BMP and TGF-β signaling pathway	(45)
	unknown	Fbxo3	Promote ubiquitination and degradation of Smurf1: promote BMP signaling pathway by stabilizing Smad1/5	(46)
	Smurf1 Lys667	unknown	Promote ubiquitination and degradation of Smurf1: promote TGF-β signaling pathway	(47)
	unknown	TRAF4	Promote ubiquitination and degradation of Smurf2: promote TGF-β signaling pathway by inhibiting degradation of TβRI	(18)
	unknown	Smad7	Promote ubiquitination and degradation of Smurf2: control resting state levels of Smurf2	(48)
	unknown	TRB3	Promote ubiquitination and degradation of Smurf2: promote TGF-β signaling pathway by stabilizing Smad3	(49)
	Smurf2 Lys48	TTC3	Promote ubiquitination and degradation of Smurf2: promote TGF-β signaling pathway by relieving degradation of Smad2/3	(50)
Neddylation	unknown	Non-covalent binding region in Smurf HECT domain	Destabilize Smurf1 on expression level	(51)
	Smurf1 Lys324/495/545 /558/559/667	UBC12	Enhance Smurf1 ubiquitin ligase activity: degrade Smad4/5, RhoA *via* ubiquitination	(52)
				
	Lys19-24 (a cluster in N-terminal region of Smurf2 HECT domain)	UBC12	Enhance Smurf2 ubiquitin ligase activity: might promote ubiquitination and degradation of RNF20?	(53)
Sumoylation	Smurf2 Lys26/369	PIAS3	Enhance Smurf2 ubiquitin ligase activity: inhibit TGF-β signaling pathway by degrading TβRI *via* ubiquitination	(54)
Methylation	Smurf2 Arg232/234/237/239	PRMT1	Reduce Smurf2 protein level: increase TGF-β-mediated reporter activity	(55)

## Phosphorylation

Protein phosphorylation is the most comprehensively studied PTM in cancers. It is a diverse process in which the phosphate group, delivered by energy carrier like Adenosine Triphosphate (ATP), covalently binds to amino acid residue (e.g., serine, tyrosine, threonine) (56) under the ordered participation of a series of molecules, such as soluble and membrane-bound extracellular chemical molecules, membrane receptors, second messengers, protein kinases (57, 58). Like other proteins, in tumor cells, Smurfs can be phosphorylated by common protein kinases [e.g., protein kinase A (PKA), PKB], and the functions of phosphorylation can be distinctive in specific tumor types. At times, phosphorylation can completely reverse the original effect of Smurfs on tumors, such as PKA inhibiting lung cancer cell growth by decreasing Smurf1 activity (38). However, at other times, phosphorylation functions as a signaling pathway to enhance positive/negative impacts of Smurfs on cancers without changing Smurfs’ original direction of action. For example, phosphorylation of Smurf1 on Thr145 is not important for DAB2IP binding, Smurf1-mediated degradation of DAB2IP, or modulation of Smad1, but it does cause Smurf1 abundance and structural stability, which further increases the degradation of DAB2IP, resulting in tumor progression (16). Phosphorylation of Smurfs by protein kinases has a plethora of implications in biological processes, including governing Smurf ubiquitin ligase activity, stability, and relative affinities for proteins, as well as mediating Smurfs’ unconventional ubiquitination of substrates, even inducing autoubiquitination and degradation of Smurfs themselves. Under diverse circumstances, phosphorylation events are closely associated with cellular processes, such as cell growth, apoptosis, proliferation, migration, and EMT (**Figures 1** and **2**).

### Smurf1 Phosphorylation

Phosphorylation of Smurf1 plays a dual role in tumor development. In different tumor types, it either boosts tumor cell progression through triggering ubiquitination and degradation of tumor suppressors [e.g., Type Iγ phosphatidylinositol phosphate kinase (PIPKIγ), DAB2IP] or induces apoptosis by promoting the abundance of corresponding factors (e.g., Nur77, RhoA) (**Figure 1**).

In some cancers, Smurf1 is phosphorylated at specific threonine residues by cAMP/PKA, which modulates Smurf1 ubiquitin ligase activity or mediates its unconventional ubiquitination of substrates. In lung cancer cells, PKA-Smurf1-PIPKIγ signal transduction plays a positive role in lung cancer cell growth and *in vivo* tumorigenesis (38). Phosphorylation of threonine residue T306 by PKA inhibits Smurf1 ubiquitin ligase activity, which abolishes Smurf1-mediated ubiquitination-dependent proteasomal degradation of PIPKIγ. PIPKIγ is reportedly highly expressed in lung cancer cells and phosphorylates and activates β-Catenin, a downstream regulator in the Wnt/Wingless signaling pathway, resulting in increased β-Catenin transcriptional activity that stimulates tumorigenic phenotypes and cell growth (40, 59). Stimulated by the chemotherapy drug cisplatin, in HeLa cells, PKA drives Smurf1 to unconventionally ubiquitinate the orphan receptor Nur77 at the K6 or K27 linkage by phosphorylation of Smurf1, which also occurrs at Thr306 (39). Conventional ubiquitination refers to the modification that leads to the degradation of the substrate proteins in the proteasome (60). For example, ubiquitination mediated by Smurf1 at Lys255 drives proteasome-dependent degradation of PIPKIγ (38). However, noncanonical ubiquitination, serving as non-degradative signaling to modulate target protein stability and localization (61), elevates protein levels of Nur77, causing subsequent Nur77 translocation into the mitochondria to induce cancer cell apoptosis (39). Interestingly, in cancer cells not treated with cisplatin, c-Jun N-terminal kinase (JNK) downregulates Nur77 (39). Nonetheless, K6 or K27 ubiquitination precludes Nur77 from degradation (39). Furthermore, phosphorylation of Smurf1 by PKA at Thr306 enhances the affinity of Smurf1 for RhoA instead of cell polarity protein Par6, accelerating degradation of RhoA *via* ubiquitination and leading to neuronal polarization (20, 62).

Akt (also called PKB), whose modulation is frequently disordered in various cancers, is a serine/threonine kinase (63). In colorectal cancer cells, phosphorylation by Akt1/2 on Smurf1 Thr145 augments Smurf1 protein stability to control DAB2IP abundance, which contributes to increased ubiquitination and degradation of tumor suppressor DAB2IP, dampening DAB2IP’s inhibitory effect on tumor cells (16). Without degradation by Smurf1, DAB2IP negatively regulates downstream pathways, but Smurf1 enhances Ras-MAPK and NF-kB oncogenic pathways by targeting DAB2IP, which bolsters tumor cell proliferation, survival, and migration (16). Interestingly, in ovarian cancer, Smurf1 also upregulates the AKT/Skp2 pathway, which further encourages cell invasiveness and EMT (64).

Other protein kinases, such as checkpoint kinase (Chk1) and Interleukin-1 Receptor Associated Kinase 2 (IRAK2), can reduce protein levels of phosphorylated Smurf1 at Thr145, Thr161, and Thr682 compared to its basic expression in cancers. In response to ultraviolet light or methyl methane-sulphonate (MMS), in HCT116 cells, phosphorylation of Smurf1 by Chk1 at Thr145, Thr161, and Thr682 motivates autoubiquitination-mediated Smurf1 degradation, leading to suppression of Smurf1-mediated degradation of RhoB. This results in RhoB accumulation and thus DNA damage-induced apoptosis (20). Due to RhoB promoting apoptosis in various cancer cells, this finding illustrates that in some cancers, Smurf1 might act as an oncoprotein to degrade RhoB and facilitate tumorigenesis (20). Meanwhile, in colorectal cancer cells, autoubiquitination and degradation of Smurf1, which is induced by ER stress stimulated IRAK2-mediated phosphorylation also at Thr145, Thr161, and Thr682, results in an altered cascade of ER effectors to inhibit cell growth and promote apoptosis (41). Therefore, upregulation of Smurf1 is associated with poor prognosis in colorectal cancer; however, the detailed mechanisms need to be further investigated (41).

### Smurf2 Phosphorylation

Among many signal cascades, TGF-β signaling is one link between Smurf2 and tumor cellular responses. It has been well established that TGF-β signaling boosts tumor cell cycle in advanced cancers (13). Smurf2 downregulates TGF-β signaling by ubiquitination degrading its primary downstream components, such as Smad2/3 and TβR (10). However, this Smurf2 function can be reversed by phosphorylation events, leading to the promotion of TGF-β signaling-mediated tumor progression (65).

HGF/c-MET signaling is positively involved in tumor development, especially in terms of cell invasion and metastasis (66). In bladder cancer cells, tyrosine kinases c-SRC and LCK, which are driven by HGF/c-MET signaling, phosphorylate Smurf2 at Tyr314/434. Smurf2 binding capacity to Smad7 is subsequently obstructed, and intramolecular C2-HECT interaction of Smurf2 is enhanced, both of which inhibit Smurf2 ubiquitin ligase activity. This action suppresses Smurf2 activity toward TβR and results in enhanced typical TGF-β signaling and TGF-β-mediated EMT, as well as cell invasion (42). This substantiates the hypothesis that HGF/c-MET needs a functional TGF-β signaling pathway for induction of tumor cell invasion and metastasis, which is realized through indirect downregulation of Smurf2 (42).

## Ubiquitination

**Figure 3:**
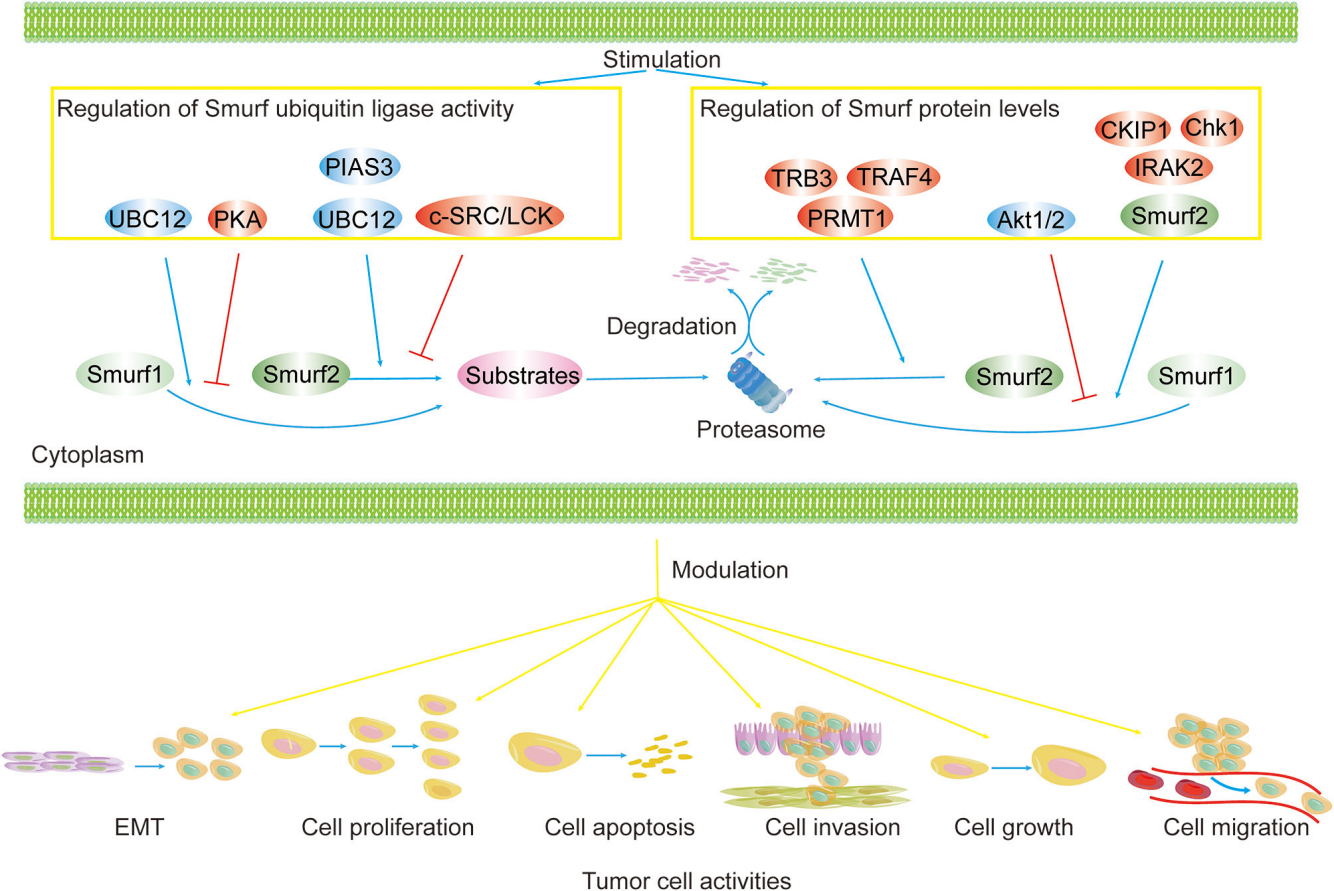
Regulation of Smurf ubiquitin ligase activity and protein levels in cancers. Smurf ubiquitin ligase activity and protein levels are tightly controlled. Smurf activity toward substrates is enhanced by activators (e.g., UBC12, PIAS3) and is repressed by inhibitors (e.g., PKA, and c-SRC/LCK). Upstream regulators (e.g., TRB3, TRAF4, PRMT1, CKIP1, Chk1, IRAK2, Smurf2) increase the degradation of Smurf1/2 *via* the proteasome pathway, yet Akt1/2 precludes Smurf1 from proteolysis. The regulation of Smurfs ultimately results in the modulation of tumor biological functions, such as cell EMT, proliferation, apoptosis, invasion, growth, and migration.

Protein ubiquitination is a common PTM defined as the covalent attachment of ubiquitin to amino acid residues of proteins, such as Lys, which is catalyzed by a defined set of enzymes (67). The ubiquitination process is achieved by the ordered action of three enzymes. Consuming energy provided by ATP, ubiquitin-activating enzyme (E1) interacts with and activates ubiquitin and then transfers ubiquitin to ubiquitin-conjugating enzyme (E2). Finally, the ubiquitin ligase (E3), in tandem with the E2 enzyme, recognizes and ubiquitinates the target protein (68). Ubiquitination events primarily trigger proteasomal degradation of proteins *via* Lys48-linked ubiquitination (61). Furthermore, ubiquitination can alter intracellular protein localization and biological activity, except for Lys48-linked ubiquitination (61). In tumor cells, ubiquitination of Smurfs mediated by regulatory proteins (e.g., Smurf2, CKIP-1, TRAF4, TRB3) aggravates the degradation of Smurfs through the proteasome pathway, leading to elevated protein levels of their substrates (e.g., RhoA, p53, Smad3) and subsequent tumor biological functions, such as cell growth, migration, and invasion, are regulated (**Figure 3**).

### Smurf1 Ubiquitination

An intermediate thioester formed by Cys699 within the C-terminus of the Smurf1 HECT domain and Gly76 of ubiquitin is pivotal to Smurf1 ubiquitin ligase activity and autoubiquitination (10, 69, 70). The thioester transfers ubiquitin to lysine residues of substrates or Smurf1 for covalent attachment, resulting in their ubiquitination (71). In tumor cells, the ubiquitination and subsequent degradation of Smurf1 changes its modulation for TGF-β-independent mechanism and thus cellular processes. For instance, downregulation of Smurf1 by Smurf2 prevents cell migration by inhibiting Smurf1-mediated mechanism independent of TGF-β signaling (43).

Smurf1 and Smurf2 exert opposite roles in modulating breast cancer progression (43). Although Smurf1 inhibits TGF-β signaling through ubiquitination and degradation of p-Smad2 in breast cancer cells, it facilitates tumor development in other ways (43). It has been reported that Smurf2 interacts with Smurf1 and exerts its ubiquitin ligase activity to ubiquitinate and degrade Smurf1, which hinders the Smurf1-mediated TGF-β-independent mechanism that leads to cell migration (43). Smurf1 is unable to directly ubiquitinate Smurf2, but it indirectly promotes ubiquitination of Smurf2 *via* mono-ubiquitinating and stabilizing TRAF4. TRAF4 facilitates poly-ubiquitination of Smurf2, leading to its degradation (18, 43). In another study, in breast cancer cells ubiquitination of Smurf1 could be reversed by the deubiquitinating enzyme USP9X through Smurf1 WW domain binding, which improved Smurf1’s stability (70). Then, Smurf1 disrupted the stability of growth-inhibitory protein RhoA by ubiquitinating and degrading RhoA, thereby promoting breast cancer cell mobility and plasticity (70). Therefore, we could conclude that Smurf1 facilitates breast cancer cell migration in a RhoA-dependent manner. In breast cancer cells, the mechanism of Smurf1 ubiquitination mediated by Smurf2, as well as Smurf1-induced mechanisms independent of TGF-β signaling, need to be further studied (43).

As a tumor suppressor, Casein kinase-2 interacting protein-1 (CKIP-1) represses Smurf1 synthesis and is responsible for autoubiquitination and degradation of Smurf1 in colon cancer cells. Then, due to low protein levels of Smurf1, the Smurf1-mediated degradation of tumor suppressor p53 is repressed, and upregulated p53 suppresses colon cancer cell growth and migration (44). The study found that Smurf1 was aberrantly expressed in colon cancer tissues, and upregulation of Smurf1 was associated with downregulation of CKIP-1 (44). However, the data showed that the protein level of Smurf1 was decreased in a concentration-dependent manner after being transfected with CKIP-1 in HCT116 cells (44). Interestingly, it has been demonstrated that a highly conserved sequence within the Smurf1 WW linker (L271NxVxCxEL279) mediates the interaction between CKIP-1 and Smurf1. Smurf2 fails to be ubiquitinated by CKIP-1 owing to a lack of this sequence (72). Surprisingly, ubiquitination induced by CKIP-1 in different types of cells, can lead to a distinct fate of Smurf1. In osteoblasts, CKIP-1 is responsible for the robust ubiquitin ligase activity of Smurf1 (72, 73). Therefore, unveiling the mechanisms of how CKIP-1 stimulating autoubiquitination of Smurf1 and thereby affecting its activity in tumor cells is a subject worthy of further researching.

F-box and leucine rich repeat protein 15 (Fbxl15) and F-box protein 3 (Fbxo3) stimulate the poly-ubiquitination and proteasomal degradation of Smurf1 by forming the SCF ubiquitin ligase complex (45, 46, 74). Through Smurf1 HECT domain binding, ubiquitination events induced by Fbxl15 at Smurf1 Lys355 and Lys357 downregulate Smurf1, which impedes Smurf1-mediated degradation of p-Smad1/5 and enhances BMP signaling (45). After Smurf1’s ubiquitination by Fbxl15, the P97-NPL4 complex formed by P97 and coenzyme nucleoprotein localization site 4 (NPL4) specifically binds to the Smurf1 PEST motif (350–373 kD) in which K355 and K357 are located and accelerates the transfer of ubiquitinated Smurf1 to the proteasome for degradation, ultimately facilitating ALK4-induced TGF-β signaling (45). This finding indicates that the P97-NPL4 complex plays a key role in Fbxl15-mediated ubiquitination and degradation of Smurf1 (46, 75). Like Fbxl15, Fbxo3 connects to Smurf1 and binds to the WW or HECT domain on Smurf1, mediating Smurf1 ubiquitination and degradation. Downregulation of Smurf1 leads to elevated protein levels of its substrates Smad1/5 and promotes BMP-2-induced BMP signaling responsiveness (46).

There are regulatory proteins (e.g., CKIP-1, Fbxo3, Fbxl15, Smurf2) that mediate ubiquitination of Smurf1. However, some factors inhibit this process. Embryonic mesoderm induction experiments found that binding of VprBP to the Smurf1-Smad7 complex decreased Smurf1 poly-ubiquitination at Lys667 (47). This action enhances Smurf1 stability, driving the Smurf1-Smad7 complex to facilitate ubiquitination and degradation of TβRI. It results in reduced TGF-β-induced phosphorylation of Smad2 and then attenuated expression of downstream target gene, thereby inhibiting embryonic mesoderm induction (47).

### Smurf2 Ubiquitination

TGF-β signaling is a bridge between the ubiquitination of Smurf2 and tumor cell activities (**Figure 2**). All of these ubiquitination events, which induce Smurf2 degradation, have a positive impact on the progression of TGF-β signaling.

Previously, we discussed that in breast cancer cells, Smurf1 boosts cell migration in a TGF-β-independent manner, which can be reversed by Smurf2 (43). However, ubiquitination of Smurf2 driven by TRAF4 in breast cancer causes Smurf2 degradation, leading to suppression of Smurf2-induced degradation of TβRI and resulting in the promotion of TGF-β cascade-induced cell migration, EMT, and invasion (18). Furthermore, deubiquitinating enzyme USP15, recruited by TRAF4, not only stabilizes TβRI by deubiquitinating TβRI but also removes ubiquitin from Smurf2 Lys734, resulting in repression of Smurf2 activity (18, 76). Deubiquitinases play a crucial role in ubiquitin-directed signaling by catalyzing the deubiquitination of substrate proteins (76). USP15 deubiquitinates Smurf2 at Lys734, a residue required for Smurf2 transthiolation and catalytic activity. Therefore, Smurf2 ubiquitin ligase activity toward TβRI is opposed by Lys734 deubiquitination, resulting in activation of TGF-β signaling (77). As mentioned above, we postulate that the negative effect of Smurf1 on TGF-β signaling is completely offset by the downregulation (in protein levels and ubiquitin ligase activity) of Smurf2, with the final result being enhanced TGF-β signaling that promotes breast cancer progression.

In TGF-β signaling, there is a feedback mechanism that governs protein levels of Smurf2 (48). In the absence of the TβR target, Smurf2 enhances its catalytic activity with respect to autoubiquitination with the help of Smad7 anchoring UbcH7 to the Smurf2 HECT domain, which leads to Smurf2 degradation and controls its resting state levels (48). However, in the presence of TβR, the binding of Smad7 with Smurf2 relieves the autoinhibition of Smurf2 by abrogating its intramolecular interaction, and this binding enhances Smurf2 ubiquitin ligase activity to inactivate TGF-β signaling (48). Furthermore, Smad7-induced autoubiquitination of Smurf2 might be another molecular mechanism of Smurf2 autoinhibition. Intramolecular interaction inhibits Smurf2 ubiquitin ligase activity, while, the Smad7-induced enhances its autoubiquitination and degradation, both of which involve the alteration of Smurf2 catalytic activity (48). It has also been indicated that three phosphorylation sites (T144 within the Smurf2 WW1 domain, T168 and T170 in C2-WW1 linker) might govern autoinhibition of Smurf2 mediated by intramolecular interactions (78). However, whether these sites regulate Smad7-Smurf2 autoinhibition remains unknown and it will broaden the horizon of regulatory mechanism of Smurf2 autoinhibition.

Tribbles homolog 3 (TRB3) and Tetratricopeptide Repeat Domain 3 (TTC3) directly ubiquitinate Smurf2, thereby mediating Smurf2 degradation in a proteasome-dependent manner. TRB3, which belongs to the pseudokinase family, is overexpressed in numerous human cell lines (49). In HepG2 cells, ubiquitination of Smurf2 by TRB3 protects Smad3 from Smurf1-mediated ubiquitination and degradation, leading to an increase in the stabilization of Smad3. Thus, the TGF-β1-Smad3 signaling pathway is augmented and activates cell migration and invasion (49). TTC3 can function as a ubiquitin E3 ligase, and its involvement in cancer cells is not well recognized (79). Poly-ubiquitination of Smurf2 by TTC3 at Lys48 relieves Smurf2-mediated degradation of Smad2/3. High protein levels of Smad2/3 lead to enhanced TGF-β signaling, which subsequently propels bronchial epithelial cell EMT (50).

## Neddylation

Similar to ubiquitination, protein neddylation is a highly ordered process (80–82). Nedd8, a highly conserved ubiquitin-like molecule containing 81 amino acid residues with 80% homology to ubiquitin (83), is conjugated to an E1 enzyme (dimer NAE composed of APPBP1 and UBA3), which then transfers Nedd8 onto an E2 enzyme (e.g., UBC12). The E3 enzyme (e.g., Rbx1, MDM2, SCFFBXO11), cooperating with the E2 enzyme, delivers Nedd8 to lysine residues on the protein. Consequently, neddylation not only controls protein steady-state levels, but also changes the localization and function of modified proteins (83–88). Neddylation events mediated by UBC12 and noncovalent binding regions within Smurfs’ HECT domain enhance Smurf ubiquitin ligase activity toward substrates and/or themselves and regulate Smurf stability (**Figure 3**). During neddylation, both Smurf1 and Smurf2 exert Nedd8 ligase activity to catalyze autoneddylation of themselves, but the regulatory mechanisms by which Nedd8 binds to Smurfs are still unclear (52, 53). Neddylated Smurfs modulate cellular processes in tumors, as shown in colorectal cancer, including cell proliferation, growth, invasion, and migration, by degrading tumor suppressors (e.g., RhoA), ultimately promoting cancer progression (**Figures 1** and **2**).

### Smurf1 Neddylation

Neddylation events driven by different factors result in activation or destabilization of Smurf1. However, effects of the noncovalent binding region on Smurf1 are complicated and multifaceted, since this region is tightly correlated with both Smurf1 neddylation and ubiquitination and may help identify novel functions of neddylation events (51), which will be discussed below.

A highly conserved Nedd8 binding sequence (L(X7)R(X5)F(X)ALQ) is discovered in the noncovalent binding region and plays a positive role in Smurf1 autoneddylation, autoubiquitination, and its ubiquitin ligase activity toward substrates, such as Smad1/5 and RhoA (51). Neddylation mediated by noncovalent binding region within the Smurf1 HECT domain destabilizes Smurf1 expression levels. When Leu, Arg, Phe, Leu, and Gln residues in the sequence were mutated to Ala (the 10A mutation), noncovalent binding of Smurf1 with Nedd8 was abolished, which partially hindered Smurf1 autoneddylation and maintained Smurf1 stability (51). In addition, Smurf1-10A selectively blocked the formation of the thioester intermediate between Smurf1 and ubiquitin, resulting in partial inhibition of Smurf1 ubiquitin ligase activity toward Smad1/5 and RhoA substrates. Upregulation of Smad1/5 and RhoA contributed to enhanced BMP signaling pathway and prevention of cell migration, respectively. Although the noncovalent binding region in Smurf1 takes a role in the promotion of cell migration, whether there is a link between neddylation of Smurf1 and cancer cell migration is unknown (51). Moreover, owing to the repression of the ubiquitin thioester intermediate formation, Smurf1-10A also suppressed Smurf1 autoubiquitination. However, 10A did not affect binding of Nedd8 to Smurf1 Cys426, both of which formed a Nedd8 thioester intermediate, since Cys426 was outside the mutated sequence (51). Smurf2-10A partially bolstered TGF-β signaling due to repression of Smurf2 ubiquitin ligase activity toward Smad3 and p-Smad3 (51). Excitingly, in view of the role of the noncovalent binding region in the neddylation and ubiquitination of Smurf1, we deem that there may be a relationship between Smurf1 neddylation and autoubiquitination in which neddylation destabilizes Smurf1 might partially by enhancing Smurf1 ubiquitin ligase activity toward autoubiquitination. However, whether exists this relationship requires further investigation.

Smurf1 has also been shown to be activated by neddylation in colorectal cancer cells. The Nedd8-thioester intermediate formed by Smurf1, Nedd8, and UBC12 (Nedd8 E2) catalyzes autoneddylation of Smurf1 at poly-lysine residues, which increases Smurf1 stability and ubiquitin ligase activity (52). Specifically, Lys324 in the Smurf1 WW-HECT linker can be mononeddylated; the first 14 lysines (in Smurf1 C2 domain) and Lys495, Lys545, Lys558, Lys559, and Lys667 (in the C-terminus of the Smurf1 HECT domain) can be multineddylated. First and foremost, the Cys426 active site within the N-lobe of the Smurf1 HECT domain is essential for the formation of Nedd8 thioester intermediate. Cys426 facilitates the transfer of Nedd8 for covalent conjugation, leading to Smurf1 autoneddylation (52). Then, through the recruitment of a ubiquitin-conjugating enzyme (E2), neddylated Smurf1 induces ubiquitination and degradation of RhoA, enhancing cell migration. Besides, Smad4/5 is also degraded *via* Smurf1 ubiquitinating (52). In this regard, the capability of Smurf1 activated by neddylation to facilitate physiological processes of colorectal cancer cells maybe also in a manner independent of TGF-β signaling. The ultimate result of this neddylation event is the acceleration of colorectal cancer cell proliferation, migration, invasion, and growth (52).

### Smurf2 Neddylation

In addition to Smurf1, Smurf2 is also subject to regulation by neddylation (53). Smurf2 interacts with Nedd8 and UBC12 to form a thioester intermediate and exerts its Nedd8 ligase activity to catalyze its autoneddylation. Neddylation enhances Smurf2 autoubiquitination and ubiquitin ligase activity toward substrates, which leads to ubiquitin-proteasomal degradation of itself and substrates respectively, by recruiting the ubiquitin-conjugating enzyme (E2) (53). Neddylation sites of Smurf2 include Lys19-24, a cluster in the N-terminal region of the Smurf2 HECT domain. Other regions within the HECT domain also have neddylation sites besides the N-terminal region. However, the covalent binding regions of Smurf2 are smaller than that of Smurf1 (52, 53). After being neddylated, Smurf2 translocates into nucleus, besides, it was previously reported that Smurf2 could maintain genome stability and decrease susceptibility to tumorigenesis by controlling RNF20 in the nucleus (33, 53). Therefore, it further noted that neddylation events might trigger Smurf2 to target RNF20 for degradation, consequently modulating chromatin conformation and tumorigenesis (33, 53). But which cancer cellular processes are affected by neddylation events is still unclear. Neddylation sites and substrates of neddylated Smurf2 in cancer cells need to be further investigated (53).

## Other Posttranslational Modifications

### Sumoylation

Protein sumoylation refers to the covalent binding of small ubiquitin-like modifiers (SUMOs) with lysine residues on proteins and occurs under the combined action of multiple enzymes, including SUMO E1 activating enzyme, SUMO-conjugating enzyme (E2), and SUMO E3 ligase (89). Like Nedd8, SUMO is also a ubiquitin-like molecule, and at this point, the sumoylation process is understood as similar to that of ubiquitination (90). After being sumoylated, protein stability, cellular distribution, activity, and interactions can be governed (91–94). In mammary epithelial cell-derived acini, sumoylation of Smurf2 by the SUMO E3 ligase (PIAS3) at Lys26 and Lys369 enhances Smurf2 stability and ubiquitin ligase activity (54). However, the sumoylation process is reversible, and SUMO molecules that covalently bind with Smurf2 can be removed by the SUMO isopeptidases SENP1/2 (54). Sumoylated Smurf2 promotes the degradation of TβRI *via* ubiquitination, restraining TGF-β signaling-mediated EMT (54). EMT is a fundamental biological process for epithelial malignant tumor cells to acquire the ability of migration and invasion (95). Thus, to further explore the effects of Smurf2 sumoylation on the progression of human breast cancer, these researchers identified that this PIAS3-sumoylated Smurf2 axis also functions as a suppressor of TGF-β signaling in human breast cancer cell-derived organoids, impairing TGF-β-induced cell invasion and growth (96). Without doubt, this novel axis lays the foundation for the development of new ideas in the treatment of breast cancer.

### Methylation

Protein methylation is well characterized and involves covalent conjunction between a methyl group and arginine or lysine residues located in protein side chains and in the N-/C-terminus (97). During this process, methyl groups are diverted by methyltransferase from methyl donors, such as S-adenosyl methionine (SAM), to substrates (98, 99). Methylation alters the properties of a protein with respect to activity, stability, positioning, and affinity for other proteins (97, 100, 101). Interestingly, the methylation process varies from one kind of residue to another. Under catalysis of arginine methyltransferase, arginine can be mono- or asymmetrically/symmetrically dimethylated. Catalyzed by lysine methyltransferase, lysine can be mono-, di- or trimethylated (97, 100). For Smurf2, protein arginine methyltransferase 1 (PRMT1), which handles more than 90% of type I protein arginine methyltransferase activity in cells, is in charge of mono- or dimethylation of internal Arg in Smurf2. Methylation of Smurf2 mediated by PRMT1 at Arg232, Arg234, Arg237, and Arg239 contributes to enhanced TGF-β-mediated reporter activity (55). Methylation does not change Smurf2 ubiquitin ligase activity, as the affinity of Smurf2 for substrates in the TGF-β signaling pathway, such as Smads and TβR, was not altered in response to methylation (55). Moreover, the cellular localization of methylated Smurf2 was also not changed relative to that of the methylation-defective mutant Smurf2 (55). Therefore, methylation events might reduce Smurf2 protein stability at the protein levels, ultimately modulating the TGF-β signaling pathway (55). Although Smurf1 shares almost 80% sequence identity with Smurf2, PRMT1 is incapable of methylating Smurf1 (55).

## Conclusions and Perspectives

Smurfs play a crucial role in numerous tumor biological functions. It is now evident that whether Smurfs promote or inhibit tumor development depends largely on tumor types and on the PTMs that they undergo, such as phosphorylation, ubiquitination, neddylation, sumoylation, and methylation (**Figures 1** and **2**). Because PTMs determine the functions of Smurfs and thus the fate of Smurf substrates, the control of Smurfs in tumor-related cellular processes is strictly and specifically modulated by upstream regulators. These upstream effects are widely applied in controlling Smurf1/2 ubiquitin ligase activity, stability (in protein levels), and interaction with other proteins, and they even mediate Smurfs’ unconventional ubiquitination of substrates (**Figure 3**). Indeed, upstream regulators vary from one kind of tumor cell to another, and it is cancer types that determine which PTMs occur (**Tables 1**, **2**). Accordingly, modification events alter Smurf management of downstream cascades, ultimately leading to promotion or inhibition of tumor progression. A typical example includes phosphorylation of Smurf1 by PKA, which inhibits Smurf1 activity and thus leads to the inhibition of Smurf1-mediated ubiquitination-dependent proteasomal degradation of PIPKIγ, resulting in lung cancer cell growth (38). Furthermore, we suspect that the occurrence of PTMs on Smurfs partially relies on sequence differences between Smurf1 and Smurf2. Although Smurf1 and Smurf2 have high structural homology, their sequence differences determine whether PTMs occur. For example, CKIP-1 mediates ubiquitination of Smurf1 rather than Smurf2 because Smurf1 contains a L271NxVxCxEL279 sequence (44). Surprisingly, to date, we also discover that there is no *in vivo* evidence of such modifications occurring in normal and cancer patient-derived tissues. Therefore, we think that in different cancer types, due to low protein levels of Smurf1/2 and/or deficiency of external stimulus of upstream factors, it makes it difficult to detect *vivo* Smurf modifications (39, 49). Meantime, modification events *in vivo* could be affected by cytokines released from other types of tissues (102). Nonetheless, *vitro* experiments of cancer cells can also reflect modification events occurring in normal and cancer tissues to a great extent (96). But we still believe that it is important to detect *in vivo* modification events, which will provide a more comprehensive molecular basis for following cancer treatment. Based on the molecular mechanisms of PTMs in Smurfs, it is of great necessity to apply targeted therapeutic molecules and drugs that target Smurfs or upstream regulatory factors to stimulate or block Smurf PTMs in order to treat cancers.

**Table 2:** The role of Smurfs in various cancer types.

Smurfs	Cancer type	Downstream protein	Promotion of biological processes	Inhibition of biological processes	Reference
Smurf1	lung cancer	PIPKIγ	/	growth, tumorigenesis	(38)
	colorectal cancer	DAB2IP	proliferation, survival, migration	/	(16)
	colorectal cancer	ER effectors	/	apoptosis	(41)
	colorectal cancer	RhoB	/	apoptosis	(20)
	colorectal cancer	RhoA	migration	/	(52)
	colon cancer	p53	growth, migration	/	(44)
	breast cancer	RhoA	migration	/	(70)
Smurf2	bladder cancer	TβR	/	EMT, invasion	(42)
	hepatoma	Smad3	/	migration, invasion	(49)
	breast cancer	TβRI	/	EMT, invasion, migration	(18)

Like other E3 ubiquitin ligases such as Mdm2, Smurf1/2 is a determinant in the genesis and/or development of some cancer types (**Table 2**), and leads to poorer survival of patients. It could be promising drug targets for cancer treatment (41). As highly conserved structures of Smurf1/2 domains, it is possible to design synthetic inhibitors such as peptides to disrupt Smurf1/2-substrate interaction (44, 103). At present, molecules targeting Smurfs are constantly appearing. Estrogen (ER), a growth factor, is significant for cell proliferation (104). Studies have shown that estrogen stimulates ERα to form a ternary complex with Smad2 and Smurf1, which induces simultaneous proteolysis of Smad2 and Smurf1 *via* ubiquitination, inhibiting the TGF-β signaling pathway (105). Researchers have also discovered drugs that activate/inactivate upstream regulators of Smurfs. It has been reported that ipatasertib and paclitaxel blocked PI3K/AKT-induced phosphorylation and activation of Smurf1 and prevented Smurf1-mediated ubiquitination and degradation of DAB2IP. As a result, DAB2IP inhibits triple-negative breast cancer cell proliferation and migration (16, 106). Moreover, in ovarian cancer, Smurf1, acting as an oncogene, facilitates cell invasiveness and EMT, partially *via* reversibly activating AKT/Skp2 signaling, and treatment with ipatasertib and paclitaxel might also exhibit efficacy in ovarian cancer (64). Cisplatin activated cAMP/PKA-mediated phosphorylation of Smurf1, leading to accumulation of Nur77 and resulting in cancer cell apoptosis (39). In another disease such as heterotopic ossification, metformin-activated AMPK induces phosphorylation and activation of Smurf1. Then, Smurf1 prevents osteoblast differentiation by degrading Runx2 (107, 108). Although metformin has been widely utilized in the treatment and prevention of cancers, so far, no relevant studies have found that it has effects on cancers through activating the phosphorylation of Smurfs (109). Owing to the limitations of these studies, clinical trials that target the molecular mechanisms of Smurf PTMs require further investigation. In this regard, significant topics, which need to be settled in future studies, include further understanding of the molecular mechanisms of Smurf PTMs and cross talk between PTMs, such as neddylation and ubiquitination. Moreover, although some PTMs (e.g., phosphorylation) are relatively well documented, the importance of others (e.g., neddylation, methylation, sumoylation) is less understood. Together, how Smurf posttranslational modifications occur, how they are terminated, and how they affect downstream pathways and cellular processes thereby determining the fate of tumors will be the focus of Smurf research for many years to come. Furthermore, breakthroughs in the molecular mechanisms of Smurf PTMs may provide new therapeutic targets for drug development.

## Author Contributions

HL, LY, and WZ conceptualized and designed the review. LY, WZ, and HL drafted the article. All authors contributed to the article and approved the submitted version.

## Funding

This work was supported by grants from the National Natural Science Foundation of China (grant number: 31900852 to HL), Natural Science Foundation of Jiangxi Province (grant numbers: 2018BAB215012 and 20192ACB21026 to HL), and Development Fund Project from Jiangxi Medical College of Nanchang University (grant number: PY201801). HL was supported by scholarship from the China Scholar Council (201906825051).

## Conflict of Interest

The authors declare that the research was conducted in the absence of any commercial or financial relationships that could be construed as a potential conflict of interest.
